# Laser acupuncture in the treatment of musculoskeletal disorders: systematic review and meta-analysis

**DOI:** 10.3389/fneur.2025.1672380

**Published:** 2026-01-12

**Authors:** Sooyoon Lee, Euijin Son, Yubin Bae, Hyo-Jin Kim, Seunghoon Lee, Jongwoo Kim, Jin-Gyun Kim, Younbyoung Chae, In-soo Jang, In-Seon Lee

**Affiliations:** 1 College of Korean Medicine, Kyung Hee University, Seoul, Republic of Korea; 2 KAIST InnoCORE PRISM-AI Center, Korea Advanced Institute of Science and Technology (KAIST), Daejeon, Republic of Korea; 3 Department of Acupuncture & Moxibustion, College of Korean Medicine, Kyung Hee University, Seoul, Republic of Korea; 4 Biomedical and Intelligent Robotics Laboratory, Department of Mechanical Engineering, Kyung Hee University, Gyeonggi-do, Republic of Korea; 5 Department of Mechanical Engineering (Integrated Engineering), Kyung Hee University, Seoul, Republic of Korea; 6 Department of Internal Medicine, College of Korean Medicine, Woosuk University, Jeonju, Republic of Korea

**Keywords:** clinical guidelines, laser acupuncture, musculoskeletal disorder, photobiomodulation, stimulation protocol

## Abstract

**Background:**

Laser acupuncture, which involves laser stimulation of acupuncture points including traditional acupoints, Ashi, and trigger points, combines the benefits of photobiomodulation and acupuncture effects. Evidence from randomized controlled trials and systematic reviews suggests that laser acupuncture and lowlevel laser therapy can reduce pain, improve functional outcomes, and decrease disability in patients with musculoskeletal disorders. However, the field is relatively new and involves complex mechanisms, leading to varied opinions on its benefits. Standardized, condition-specific stimulation protocols for laser acupuncture have not yet been established, as existing studies report heterogeneous parameters and lack consistent reporting practices. One major challenge in validating the clinical effects of laser acupuncture is the inconsistency in nomenclature and the lack of consensus on critical parameters and clinical guidelines.

**Objective:**

This study aims to provide a descriptive synthesis of existing clinical evidence on laser acupuncture for musculoskeletal disorders and to describe the range and reporting quality of laser-related parameters used in these studies. In doing so, it offers a basis for future efforts to harmonize reporting and clinical practice.

**Methods:**

We reviewed 28 randomized controlled trials focused on laser acupuncture for musculoskeletal disorders and conducted a meta-analysis on 14 of these studies. Key variables included laser type, wavelength, mode, duration, frequency, irradiance, power density, area, density/probe, total exposure energy, exposure time, treatment frequency and duration, and clinical outcomes.

**Results:**

Our findings revealed that many studies did not distinguish terms like Low-Intensity Laser Therapy from laser acupuncture, and lacked detailed descriptions of laser parameters, which could affect outcomes. The complexity of laser acupuncture mechanisms and its diverse variables make it challenging to understand which factors impact therapeutic effects.

**Conclusion:**

Therefore, it is crucial to detail all possible variables in future research to clarify the relationship between dosage and treatment effects. Finally, due to challenges in applying current guidelines, new guidelines specifically for laser acupuncture research may be necessary.

**Systematic review registration:**

https://osf.io/es9k2.

## 1 Introduction

Musculoskeletal disorders (MSDs) are a leading cause of pain, disability, and productivity loss worldwide, imposing a substantial clinical and socioeconomic burden on patients and health systems (1). A recent global macroeconomic analysis estimated that MSDs account for approximately 1.4% of global gross domestic product in lost economic output, highlighting their considerable economic impact (2).

In parallel with this growing socioeconomic burden, there has been a policy-driven shift toward conservative and non-invasive management of musculoskeletal disorders in recent healthcare frameworks (3, 4). Recent healthcare transformation efforts in Hong Kong emphasize prevention-focused, primary care strategies to address musculoskeletal health needs and reduce downstream healthcare costs associated with chronic pain conditions, reflecting a broader movement toward early, non-pharmacologic intervention (4). Similar policy directions are evident in mainland China, where the Healthy China Initiative 2030 promotes noninvasive and nonpharmacological strategies to prevent and manage chronic musculoskeletal problems as part of a national agenda prioritizing health promotion and disease prevention (3). Non-invasive management of musculoskeletal conditions has also been widely recommended in international health policy contexts, including World Health Organization–aligned frameworks, as a means of reducing reliance on invasive procedures and medication-based care (5).

Within these conservative approaches, laser acupuncture has emerged as a potential non-pharmacological option. Laser acupuncture stimulates acupoints via photobiomodulation and may reduce musculoskeletal pain through mitochondrial activation and related cellular mechanisms (6). Laser acupuncture integrates low-level laser therapy (LLLT) with traditional acupuncture principles. It can be administered in both invasive and non-invasive forms, and can be broadly understood as a form of photobiomodulation applied to acupuncture points (7).

Low-level laser therapy is a therapeutic modality that involves the application of low-intensity laser light to biological tissues (6). LLLT stimulates biological responses at the cellular level by using light of specific wavelengths, promoting pain relief, reducing inflammation, and aiding tissue regeneration. As the laser penetrates the skin, scattering and absorption occur, which vary depending on the laser’s wavelength, frequency, and power (8). Human skin comprises multiple layers with heterogeneous tissue orientations and compositions, resulting in complex optical properties. Consequently, the same treatment may yield different effects depending on the patient-specific characteristics (56).

Laser acupuncture, as LLLT, has been utilized in managing pain, treating inflammation, and promoting tissue regeneration across various medical conditions Laser acupuncture, as LLLT, has been utilized in managing pain, treating inflammation, and promoting tissue regeneration across various medical condition (9). The key clinical effects may include anti-inflammatory responses, neuromodulation, and activation of cell-signaling pathways (10). Previous studies have demonstrated that red and near-infrared LLLT produces anti-inflammatory effects similar to those of non-steroidal anti-inflammatory drugs (11). Experimental and preclinical studies further suggest that laser stimulation of acupuncture points may modulate pain processing at both peripheral and central levels, attenuate somatosensory responses to noxious stimuli, and enhance neuronal survival in models of neurodegeneration, indicating potential neuroprotective and neuromodulatory effects (13, 54), and enhanced ATP production (12, 55). Collectively, these findings imply that laser acupuncture may exert therapeutic benefits through photobiomodulation-driven modulation of inflammatory and neural pathways, although the precise mechanisms underlying these effects remain to be fully elucidated. Clinical studies have similarly reported the effectiveness of laser acupuncture in relieving pain and improving function in chronic neck pain, low back pain, and knee osteoarthritis. However, most of these studies have relatively small sample sizes and heterogeneous stimulation parameters, making it difficult to draw firm conclusions from individual studies alone.

To synthesize the available evidence, a meta-analysis including 49 randomized controlled trials (RCTs) evaluating pain as a primary outcome reported positive effects of laser acupuncture, showing favorable outcomes in pain reduction compared to placebo interventions at the end of treatment and during the follow-up period. Subgroup analyses for myofascial pain, musculoskeletal trigger points, lateral epicondylitis, and temporomandibular joint pain also demonstrated effectiveness both in the short- and long-term periods (9). Collectively, these studies suggest that laser acupuncture may be a useful non-pharmacological option for managing musculoskeletal pain.

However, the overall certainty of this evidence remains limited because many studies have small sample sizes and methodological weaknesses, and there is considerable variability in laser parameters, treatment protocols and outcome measures (14). Although a number of randomized controlled trials and reviews support the potential effectiveness of laser acupuncture for musculoskeletal pain, they rarely prioritize laser parameters and dosage as key analytical variables. As a result, there is still no clear, empirically derived framework for selecting or reporting parameter combinations (e.g., wavelength, power, energy density and total dose) in clinical trials of laser acupuncture, and important questions—such as which musculoskeletal conditions benefit most from laser acupuncture and which parameter combinations and dosages are most appropriate—remain insufficiently addressed.

To support both mechanistic and clinical research, comprehensive investigation of laser parameters and detailed reporting of treatment characteristics, including laser dosage, are essential. Nonetheless, awareness and reporting of key laser-related parameters (e.g., power and irradiance), as well as treatment conditions and environments, remain limited, which complicates efforts to evaluate the clinical effectiveness of laser acupuncture and to develop standardized treatment protocols. This heterogeneity, together with limited understanding of how laser parameters influence both clinical outcomes and physiological changes, constrains our ability to clarify the relationship between laser acupuncture dosage and therapeutic effects.

The Standards for Reporting Interventions in Clinical Trials of Acupuncture (STRICTA) guidelines were developed to enhance the reproducibility and reliability in acupuncture research (15), but they do not completely capture the laser-specific parameters. More detailed reporting of laser-specific parameters is therefore needed to improve transparency and consistency in future studies.

To address these issues, this study aims to provide a descriptive synthesis of existing RCTs of laser acupuncture for musculoskeletal disorders and to investigate the following: (1) how laser acupuncture has been administered in clinical studies treating musculoskeletal disorders; (2) the extent and quality of reporting of key laser-related variables; and (3) reporting gaps and considerations that may inform the future development of standardized protocols and reporting guidelines for laser acupuncture studies. To achieve these objectives, we conducted a systematic review and meta-analysis of randomized controlled clinical trials using laser acupuncture for patients with musculoskeletal disorders.

## 2 Methods

This study was designed as a systematic review and meta-analysis of RCTs evaluating laser acupuncture for musculoskeletal disorders. The review methods were defined *a priori* in a protocol that was registered with the Open Science Framework (https://osf.io/es9k2) and conducted in accordance with the Preferred Reporting Items for Systematic Reviews and Meta-Analyses (PRISMA) 2020 guidelines. Based on the aims described at the end of the Introduction, the review proceeded in two steps: First, we summarized potential dosage-related parameters in RCTs on the effects of laser acupuncture for musculoskeletal disorders, involving a systematic review and thorough examination of current literature. Second, we conducted a meta-analysis of pain-related outcomes to understand the effects of various dosages and evaluate the overall efficacy of laser acupuncture.

### 2.1 Literature search

We conducted the literature search in accordance with PRISMA 2020. The final search was performed on 22 September 2023 using PubMed. The search strategy combined laser-related terms (e.g., “laser,” “low-level laser therapy,” “LLLT,” “low-intensity laser therapy,” “photobiomodulation”) with musculoskeletal- and pain-related terms (e.g., “musculoskeletal,” “musculoskeletal disorders,” “back pain,” “neck pain,” “knee osteoarthritis,” “joint pain,” “myofascial pain,” “chronic pain”) and study design terms (“randomized controlled trial,” “RCT”). Both free-text keywords and Medical Subject Headings (MeSH) were used where available. The electronic search was performed independently by two reviewers (SYL and EJS). One reviewer (SYL) screened titles and abstracts and made initial inclusion and exclusion decisions, which were checked for consistency by a second reviewer (EJS). The same two reviewers then assessed the full texts of potentially eligible studies. Data extraction was carried out by SYL and then cross-checked by EJS. Any disagreements at the study selection or data extraction stages were resolved through discussion, and when necessary by consultation with a third reviewer (ISL). The exclusion criteria included studies not written in English, non-RCTs, and studies unrelated to laser acupuncture and musculoskeletal disorders. No limits on publication year were applied, and only peer-reviewed articles were included.

### 2.2 Eligibility criteria and study selection

We included published RCTs that evaluated laser acupuncture in human participants with musculoskeletal disorders or musculoskeletal pain. Trials were eligible regardless of specific musculoskeletal condition, age, sex, or clinical setting, and could include healthy participants if musculoskeletal pain or musculoskeletal outcomes were assessed. We required that laser stimulation was applied to acupuncture points (invasive or non-invasive), either as a standalone intervention or in combination with other treatments, and that the study reported at least one pain-related or musculoskeletal clinical outcome. We excluded non-randomized studies, case reports and case series, animal or *in vitro* studies, conference abstracts without full text, articles not written in English, studies that did not use laser acupuncture, and studies that were not related to musculoskeletal disorders.

### 2.3 Data extraction and data items

The primary outcomes focused on laser-related parameters (see Supplementary Table 1 for more details), such as pulse, power, irradiance, total energy, energy density, duration, and frequency of laser application. Secondary outcomes summarized details of the treatment (e.g., invasiveness, stimulation sites, and number of points used) and study design (e.g., study type, population, and sample size). We compiled details of acupuncture interventions (e.g., acupuncture style, other interventions, number of needles per session, acupuncture points used, depths of insertion, deqi response, stimulation degree, needle retention time, needle information, and treatment session parameters) according to the STRICTA guidelines (15). Two reviewers (SYL and EJS) independently screened articles based on titles, abstracts, and full texts for eligibility according to the inclusion criteria. We recorded the number of articles selected and rejected along with the reasons for each decision at each step. Data were independently extracted from selected articles by the two reviewers and cross-checked for accuracy. Disagreements were resolved through discussions between the two reviewers and, if necessary, with a third reviewer (ISL), a doctor of Korean Medicine specialized in the theory of meridian and acupoints.

As laser-specific parameters such as laser manufacturer, type, mode, pulse, power, irradiance, and energy density are not addressed in the existing version of the STRICTA guidelines, we suggested a new guideline for reporting laser acupuncture. By incorporating detailed parameter information, the proposed parameter guidelines can standardize reporting for laser acupuncture research, potentially enhancing the comparability and reproducibility of research outcomes.

### 2.4 Meta-analysis

We conducted a meta-analysis of the effects of laser acupuncture measured using either Visual Analog Scale (VAS) or the Numerical Rating Scale (NRS) of pain ratings. Studies that did not report pain intensity, or used alternative method (such as improvement of pain and pain-related questionnaire scores), were excluded from the meta-analysis. Comparators included either a sham intervention or other treatments, as we included trials comparing laser acupuncture plus another intervention to the another intervention alone. We calculated the effect size for mean differences between groups using a pooled pre-test standard deviation for a pre-post design [dppc2 in (16)]. The meta-analysis utilized Meta-Essentials for continuous data between independent groups (55). We applied a random effects model, used a 95% confidence level to calculate the combined effect size, and generated a forest plot. We also assessed heterogeneity (I2 index) and publication bias using Egger’s regression test (17) and the Begg-Mazumdar rank correlation test (18). Statistical significance was defined as a *p*-value < 0.05.

### 2.5 Risk of bias assessment

We evaluated the risk of bias for each study using the revised Cochrane Risk of Bias Tool for Randomized Trials (RoB 2.0) (19). Details and results are provided in the Supplementary Table 2.

### 2.6 Certainty of evidence

In addition to assessing risk of bias using the RoB 2.0 tool, we qualitatively appraised the certainty of the evidence for pain outcomes using domains analogous to the GRADE framework, including risk of bias, inconsistency, imprecision, indirectness, and publication bias. We did not construct a formal GRADE evidence profile, but we summarized certainty narratively based on these criteria.

## 3 Results

### 3.1 Results of the search

**Figure 1:**
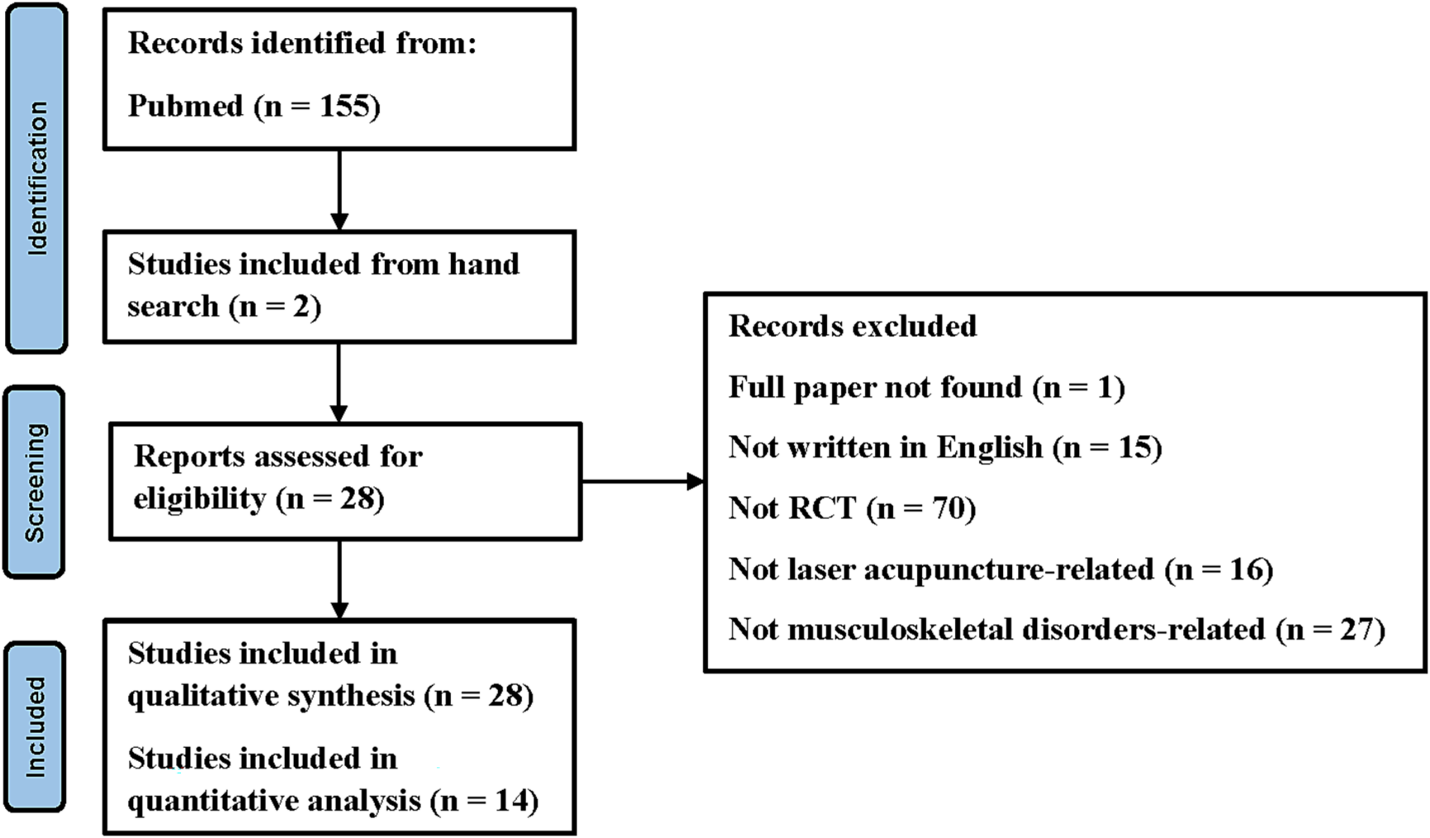
Flowchart of the study selection process for systematic review and meta-analysis.

A flowchart based on the PRISMA guidelines is shown in Figure 1. A total of 155 studies were identified through electronic searches and two additional papers were identified through manual searching. After screening, 28 studies were included in the qualitative synthesis, while 14 studies were included in the quantitative analysis (not written in English *n* = 15; not RCTs, *n* = 70; not laser acupuncture used *n* = 16; not musculoskeletal disorders-related *n* = 27; full text not available *n* = 1).

### 3.2 Systematic review of included RCTs

The systematic review included studies published between 1989 and 2023, comprising 28 RCTs with a total of 2,021 participants. Thirteen studies used continuous-wave laser acupuncture, while eight studies used pulsed laser acupuncture (seven studies were unclear about the type). Of the 28 RCTs, 14 studies that reported pain scores measured using the VAS or the NRS were included in the meta-analysis. Since three out of these 14 studies featured more than one comparison, a total of 18 comparisons were included in the meta-analysis.

Sample sizes of individual studies ranged from 20 to 392 participants. The most commonly investigated condition was knee osteoarthritis (9 trials), followed by low back pain including non-specific chronic low back pain (5 trials), temporomandibular disorders (3 trials), delayed-onset muscle soreness (2 trials), cervical or generalized myofascial pain (4 trials) and other musculoskeletal conditions such as whiplash injuries, subacromial impingement syndrome, lateral epicondylalgia, fibromyalgia and wrist fracture.

#### 3.2.1 Laser-related parameters of laser acupuncture

Among the 28 studies, only one involved invasive laser acupuncture, where the laser was inserted 4 cm into the body. Twenty-six studies stimulated acupoints, including ashi points, while five targeted triggers or tender points. Sixteen studies referred to the ‘laser acupuncture,’ 9 studies as ‘LLLT,’ 4 studies as ‘laser therapy/treatment,’ and 2 studies each used the terms ‘LILT’ and ‘laser moxibustion.’ Three studies emphasized that laser treatments were applied to acupuncture points. Fourteen studies employed GaAlAs lasers, seven did not specify the type of laser used, three utilized CO2 lasers (sometimes in combination with other lasers), two studies used GaAs lasers, and one study each used HeNe, diode, and Neodymium: YAG lasers (Table 1).

**Table 1:** Characteristics and laser-related parameters of eligible randomized clinical trials.

Author (year)	Study design	Details of LA	Treatment regimen	Outcomes	Expressions used for laser	Device information	Device setting	Dose
	1. Type of study 2. Included population 3. Size of study	1. Invasiveness 2. Stimulation sites 2a. Acupoints 2b. Non-acupoints 3. Number of points used	1. Frequency of treatment sessions 2. Duration of treatment sessions	1. Pain-related 2. Others		1. Manufacturer 2. Type of laser 3. Laser mode	1. Pulse 2. Power 3. Irradiance	Continous-wave 1. Area/point 2. Total energy 3. Energy density 4. Duration (only for pulsed laser) 5. Pulse duration 6. Frequency 3. Duty cycle
Continuous-wave laser								
Aigner et al. (26)	1. Prospective RCT 2. Whiplash injuries 3. *n* = 50 (analysis *n* = 45) (1) LA (*n* = 25) (2) Placebo LA (*n* = 25)	1. No 2a. BL10, BL40, GB20, GB34, SJ5, SI6, GV14 Ear points 29, 37, 41, 55 (NR) 2b. No 3. NR	1. 3/wk. 2. 3 wks	1. Neck pain, headaches, pain duration 2. ROM, dizziness	LLLT	1. Silberbauer 2. HeNe 3. Continuous	1. 632.8 nm 2. 5 mw 3. NR	1. NR 2. NR 3. 0.075 J/cm^2^ 4. 15 s/point
Mazzetto et al. (27)	1. Double-blinded RCT 2. TMD 3. *n* = 48 (analysis *n* = 48) (1) LILT (*n* = 24) (2) placebo (*n* = 24)	1. No 2a. Acupoints in the external auditive duct toward retrodiskal region (Bi) 2b. No 3. 2	1. 2/wk. 2. 4 wks	1. VAS 2. –	LILT	1. MM Optics 2. GaAlAs 3. Continuous	1. 780 nm 2. 70 mW 3. NR	1. NR 2. NR 3. 89.7 J/cm2 4. 10 s/point
Ferreira et al. (28)	1. Double-blinded, prospective, placebo-controlled RCT 2. TMD 3. *n* = 53 (analysis *n* = 40) (1) NMOS + LA (*n* = 20) (2) NMOS + placebo (*n* = 18)	1. No 2a. ST6, SI19, GB20, GB43, LI4, LR3, TE3, GB34, EX-HN3 (NR) 2b. No 3. NR	1. 1/wk. 2. 12 wks	1. VAS 2. RDC/TMD application	LA	1. MM Optics 2. GaAlAs 3. Continuous	1. 780 nm 2. 50mw 3. 1,250 w/cm^2^	1. 0.04 cm^2^ 2. NR (4.5 J/point) 3. 112.5 J/cm^2^ 4. 90 s/point
Al Rashoud et al. (29)	1. Double-blinded RCT 2. Knee osteoarthritis 3. *n* = 58 (analysis *n* = 49) (1) Active laser (*n* = 26) (2) Placebo laser (*n* = 23)	1. No 2a. SP9, SP10, ST35, ST36, EX-LE4 (Uni) 2b. No 3. 5	1. 3/wk. 2. 3 wks	1. VAS 2. SKFS, ROM	LLLT	1. Enraf Nonius 2. GaAlAs 3. Continuous	1. 830 nm 2. 30mw 3. NR	1. 0.28cm^2^ 2. 6 J 3. 4 J/cm^2^ 4. 40 s/point
Fleckenstein et al. (30)	1. Double-blinded RCT 2. DOMS 3. *n* = 60 (1) Verum acupuncture (*n* = 12) (2) sham acupuncture (*n* = 12) (3) LA (*n* = 12) (4) Sham LA (*n* = 12) 5) no treatment (*n* = 12)	1. No 2a. LI4, LI11, LU3, LU5, GB34, SP10, ashi points (NR) 2b. TP, individualized points 3. Various	1. 1/day 2. 3 days	1. VAS, PPT 2. Mean isometric voluntary force	LA	1. 3B Scientific 2. NR 3. Continuous	1. NR 2. NR 3. NR	1. NR 2. NR 3. NR 4. NR
Kibar et al. (31)	1. Double-blinded, sham controlled RCT 2. Subacromial impingement syndrome 3. *n* = 73 (analysis *n* = 62) (1) Exercise training + LA (*n* = 30) (2) Exercise training + sham LA (*n* = 32)	1. No 2a. GB21, LI4, LI11, LI14, LI15, LI16, SI9, SI10, SI11, TE14, TE15 (NR) 2b. No 3. 11	1. 5/wk. 2. 3 wks	1. VAS, shoulder pain and disability Index 2. Shoulder ROM	LA, LILT on acupuncture points	1. Chattanooga Group 2. GaAlAs 3. Continuous	1. 850 nm 2. NR 3. NR	1. 0.07 cm^2^ 2. 40 J 3. 4 J/cm^2^ 4. 40 s/point
Mohammed et al. (32)	1. Single-blinded, prospective RCT 2. Knee osteoarthritis 3. *n* = 40 (analysis *n* = 40) (1) LA (*n* = 20) (2) Sham laser (*n* = 20)	1. No 2a. ST35, ST36, SP9, SP10, GB34 (NR), ashi points 2b. No 3. NR	1. 3/wk. 2. 4 wks	1. VAS 2. X-ray, laboratory tests	LA	1. Petrolaser 2. GaAlAs 3. Continuous	1. NR 2. 90mw 3. 2.8w/cm^2^	1. 2 mm 2. NR 3. 5.4 J (acupoints) 21.6 J (ashi point) 4. 1 min (acupoints) 4 min (ashi points)
Madani et al. (33)	1. Double-blinded RCT 2. TMD 3. *n* = 45 (analysis *n* = 45) (1) LLLT for Muscles/joints (*n* = 15) (2) LA (*n* = 15) (3) Placebo (*n* = 15)	1. No 2a. ST6, ST7, LI4 (Bi) 2b. masticatory muscles/TMJ 3. 6	1. 2/wk. 2. 5 wks	1. VAS 2. Mandibular ROM, amount of mouse opening	LLLT, LA	1. Thor 2. GaAlAs 3. Continuous	1. 810 nm 2. 200mw 3. NR	1. 0.28cm^2^ 2. 6 J 3. 21 J/cm^2^ 4. 30 s/point
Liao et al. (34)	1. Double-blinded RCT 2. Knee osteoarthritis 3. *n* = 33 (analysis *n* = 30) (1) Active laser (*n* = 15) (2) placebo laser (*n* = 15)	1. No 2a. SP9, SP10, EX-LE2 (Bi) 2b. No 3. 6	1. 3/wk. 2. 4 wks	1. VAS 2. Lequesne index	LLLT	1. TI-816-2, Transverse 2. NR 3. Continuous	1. 780, 830 nm 2. 50 mW (780); 30 mW (830) 3. NR	1. NR 2. 216 J 3. NR 4. 15 min
Kholoosy et al. (35)	1. Single-blinded RCT 2. LBP 3. *n* = 40 (analysis *n* = 34) (1) Laser (*n* = 19) (2) sham laser (*n* = 15)	1. Yes (4 cm) 2a. LI4, ST44, HT7 (NR) 2b. articular spaces of vertebral column, adjacent paravertebral points, pain radiating areas, TP 3. Various	1. 3/wk. 2. 4 wks	1. VAS 2. RMDQ, TP	LLL(T)	1. Canadian Optic and Laser Center 2. GaAlAs 3. Continuous	1. 808 nm 2. 160 mw 3. NR	1. 1cm^2^ 2. 4.5 J/point 1.5 J/meridian 3. 0.16 J/cm^2^ 4. 10 s/point (meridians); 30 s/point (others)
Chang et al. (36)	1. Single-blinded RCT 2. Cervical myofascial pain syndrome 3. *n* = 100 (analysis *n* = 100) (1) Acupoints (*n* = 25) (2) Control acupoint (*n* = 25) (3) TP (*n* = 25) (4) Control TP (*n* = 25)	1. No 2a. (1) LI10, LI4, TE5, SI3 (Uni) 2b. (3) TP 3. 4	1. 1 2. 1	1. VAS, PPT 2. Cervical ROM	LLLT	1. Reimers and Janssen gmbh 2. GaAlAs 3. Continuous	1. 810 nm 2. 150 mw 3. 0.76 w/cm^2^	1. 0.5 cm 2. 8 J 3. NR 4. (1) 40 s (3) 160 s
Fang et al. (37)	1. RCT 2. Knee osteoarthritis 3. *n* = 92 (analysis *n* = 85) (1) Laser moxibustion (*n* = 42) (2) Traditional moxibution (*n* = 43)	1. No 2a. ST35, ashi points (NR) 2b. No 3. Various	1. 3/wk. 2. 4 wks	1. VAS, WOMAC pain 2. Walking time test	Laser moxibustion	1. Shanghai Wonderful Opto-Electrics Tech 2. NR 3. Continuous	1. 10.6 μm 2. 170 mW 3. NR	1. 2 cm 2. NR (203.91 J/point) 3. 64.97 J/cm^2^ 4. 20 min
Sajedi et al. (38)	1. Double-blinded RCT 2. Myofascial pain syndrome 3. *n* = 60 (analysis *n* = 60) (1) low-level LA (*n* = 30) (2) cupping (*n* = 30)	1. No 2a. NR 2b. TP in masticatory muscle 3. Various	1. 1/day 2. 8 sessions	1. VAS 2. painless maximum mouth opening, patient satisfaction, number of TP, number of treatment sessions required until recovery	Low-level LA	1. Konftec 2. GaAlAs 3. Continuous	1. 808 nm 2. 0.5 W 3. NR	1. NR 2. 30 J 3. 4 J/cm^2^ 4. 60 s/point
*Pulsed laser*								
Ceccherelli et al. (39)	1. Double-blinded RCT 2. Cervical myofascial pain syndrome 3. *n* = 27 (1) Laser therapy (*n* = 13) (2) Placebo laser therapy (*n* = 14)	1. No 2a. LI4, LI11, LI14 SI3, TE5 (Bi), individualized points 2b. TP 3. 14	1. 3/wk. 2. 4 wks	1. VAS, McGill pain questionnaire 2. -	Laser therapy	1. Nuova Vitiemme R 2. Diode laser 3. Pulse	1. 904 nm 2. 25 W 3. NR	1. NR 2. 5 J 3. NR 4. NR 5. 200 ns 6. 1,000 Hz 7. 0.75
Haker and Lundeberg (40)	1. Double-blinded RCT 2. Lateral humeral epicondylalgia 3. *n* = 49 (analysis i = 40) (1) Laser (*n* = 18) (2) Placebo laser (*n* = 22)	1. No 2a. LI10, LI11, LI12, LU5, TE5 (NR) 2b. No 3. -	1. 2-3/wk. 2. 3–5 wks	1. Pain 2. Resisted wrist extension, stretching of the extensor muscle, middle finger test, resisted pronation/supination, vigorimeter test, lifting test	Laser treatment applied to acupoints	1. Mid 1,500 IRRADIA 2. GaAs 3. Pulse	1. 904 nm 2. 12mw 3. NR	1. NR 2. NR 3. 0.36 J/point. 4. 30 s/point 5. NR 6. 70 Hz 7. NR
Yurtkuran et al. (41)	1. Double-blinded RCT 2. Knee osteoarthritis 3. *n* = 55 (analysis *n* = 52) (1) LLLT (*n* = 27) (2) Placebo laser (*n* = 26)	1. No 2a. SP9 (Uni) 2b. No 3. 1	1. 5/wk. 2. 2 wks	1. VAS 2. WOMAC, 50 foot walking time, medial tenderness score, quality of life, knee circumference	LA, LLLT	1. Roland Serie Elettronica Pagani 2. GaAs 3. Pulse	1. 904 nm 2. 4 mw 3. 10 mw/cm^2^	1. 0.4 cm2 2. 0.48 J 3. NR 4. 2 min 5. 200 ns 6. NR 7. NR
Shen et al. (42)	1. RCT 2. Knee osteoarthritis 3. *n* = 40 (1) LA (*n* = 20) (2) placebo LA (*n* = 20)	1. No 2a. ST35 (Uni/Bi) 2b. No 3. 1 or 2	1. 3/wk. 2. 4 wks	1. WOMAC pain 2. WOMAC stiffness/function, global assessment, adverse events, medication usage	LA	1. self-made 2. GaAlAs+CO2 3. Pulse	1. 10.6 μm (GaAlAs) 650 nm (CO2) 2. 36 mw (GaAlAs) 200 mw (CO2) 3. NR	1.2 mm 2. NR 3. NR 4. 20 min 5. NR 6. 40 Hz (CO2) 7. 50% (CO2)
Zhao et al. (43)	1. RCT 2. Knee osteoarthritis 3. *n* = 40 (analysis *n* = 31) (1) LA (*n* = 18) (2) sham LA (*n* = 13)	1. No 2a. ST35 (Uni/bi) 2b. No 3. 1 or 2	1. 3/wk. 2. 4 wks	1. WOMAC pain 2. WOMAC stiffness/function, global assessment, medication usage, masking effectiveness, adverse effect	LA	1. NR 2. CO2 3. Pulse	1. 650 nm 10.6 mm 2. 36 mw 200 mw 3. NR	1. 2 mm 2. 163.2 J 3. NR 4. 20 min 5. NR 6. 40 Hz 7. 50%
Lin et al. (44)	1. RCT 2. LBP 3. *n* = 60 (analysis *n* = 42) (1) LA + soft cupping (*n* = 21) (2) sham LA + soft cupping (*n* = 21)	1. No 2a. BL40 (Bi), ashi points 2b. No 3. various	1. 1/day 2. 5 days	1. VAS 2. Ryodoraku value	LA	1. United integrated services 2. NR 3. Pulse	1. 808 nm 2. 40mw 3. NR	1. 0.8 cm2 2. NR 3.15 J/cm2 4.10 min 5. NR 6. 20 Hz 7. 50%
Chang et al. (45)	1. Triple-blinded RCT 2. DOMS 3. *n* = 40 (1) LA (*n* = 20) (2) placebo LA (*n* = 20)	1. No 2a. PC2, LU5 (Bi) 2b. No 3. 2	1. 1/day 2. 1 days	1. VAS, PPT 2. arm circumference, muscle strength	LA	1. Advanced Chips and Products Crop 2. NR 3. Pulse	1. 830 nm 2. 60mw 3. NR	1. NR 2. 36 J 3. 9.7 J/cm2 4.10 min/point 5. 0.75 ms 6. 10 Hz 7. NR
Boggiss et al. (46)	1. Pilot RCT 2. Fibromyalgia 3. *n* = 20 (analysis *n* = 16) (1) LA (*n* = 9) (2) education (*n* = 7)	1. No 2a. LU9, PC6, HT7, LI5, TE4, SI5, SP3, LR3, KI4, BL65, GB40, ST42 (bi) 2b. No 3. Various	1. 2/wk. 2. 3 wks	1. NRS, generalized pain index, symptoms severity scale 2. Heart rate variability	LA	1. Ecco fibras 2. NR 3. Pulse	1. 808 nm 2. 120mw 3. NR	1. NR 2. NR 3. 5 J/cm2 4. Various 5. NR 6. 36.5 Hz 7. NR
*Unclear*								
Glazov et al. (47)	1. Double-blinded prospective RCT 2. LBP 3. *n* = 100 (analysis *n* = 90) (1) LA (*n* = 45) (2) Sham laser (*n* = 45)	1. No 2a. BL, GB, GV, distal acupoints, ashi points (NR) 2b. TP 3. NR	1. 1/wk. 2. 5 ~ 10 wks	1. VAS 2. ODI, global assessment, depression anxiety stress scale, subjective wellbeing, exercise, analgesic use, adverse effects	LA	1. Acupak 2. GaAlAs 3. NR	1. 830 nm 2. 10 mW 3. 0.05 W/cm^2^	1. NR 2. NR 3. 0.2 J/point 4. 20 s/point
Glazov et al. (48)	1. Double-blinded, prospective, three group parallel RCT 2. Non-specific chronic LBP 3. *n* = 144 (analysis *n* = 127) (1) low-dose LA (*n* = 42) (2) high-dose LA (*n* = 40) (3) Sham LA (*n* = 45)	1. No 2a. individualized points (NR) 2b. No 3. 9 (average)	1. 1/wk. 2. 8 wks	1. NRS 2. ODI, limitation of activities, global assessment, frequency of analgesics intake, adverse effects	Low-dose LA, LLLT	1. Acupak 2. GaAlAs 3. NR	1. 830 nm 2. 20 mW 3. 0.1 W/cm^2^	1. X 2. X 3. (1) 0.2 J/point (2) 0.8 J/point 4. (1) 10s (2) 40s
Helianthi et al. (49)	1. Double-blinded RCT 2. Knee osteoarthritis 3. *n* = 62 (analysis *n* = 59) (1) Active LA (*n* = 30) (2) placebo LA (*n* = 29)	1. No 2a. ST35, ST36, SP9, GB34, EX-LE4 (NR) 2b. No 3. NR	1. 2/wk. 2. 5 wks	1. VAS 2. Lequesne index	LA	1. Handylaser Trion RJ 2. GaAlAs 3. NR	1. 785 nm 2. 50 mW 3. 25 mW/cm^2^	1. NR 2. 4 J/point 3. NR 4. 80 s/point
Acosta-Olivo et al. (50)	1. Double-blinded RCT 2. Wrist bone fracture 3. *n* = 26 (1) Rehabilitation + LA (*n* = 13) (2) Rehabilitation + control laser (*n* = 13)	1. No 2a. SI5, TE4, TE15, LI5, PC7, BL62, BL60, KI3 (Uni), LI4 (Bi) 2b. No 3.10	1. 3/wk. 2. 10 sessions	1. VAS 2. PRWE	Laser treatment on acupoints	1. Diller and Diller Laser Performance 2. NR 3. NR	1. 980 nm 2. 50 mW 3. NR	1. NR 2. NR 3. NR 4. 30 s/point 5. NR 6. 8,000 Hz 7. NR
Zhao et al. (51)	1. Multi-site, double-blinded RCT 2. Knee osteoarthritis 3. *n* = 392 (analysis *n* = 364) (1) Laser moxibustion (*n* = 191) (2) sham control (*n* = 173)	1. No 2a. ST35 (Uni/Bi), ashi points 2b. No 3. NR	1. 3/wk. 2. 4wks	1. VAS, WOMAC pain 2. SF36, global assessment, WOMAC stiffness/physical function	Laser moxibustion	1. Shanghai Wonderful Opto -electrics Tech. 2. CO2 3. NR	1. 10.6 μm 2. 160–180 mW 3. NR	1. NR 2. NR 3. 61.2–68.8 J/cm^2^ 4. 20 min
Ahi and Sirzai (52)	1. Single-blinded RCT 2. Myofascial pain syndrome 3. *n* = 108 (analysis *n* = 105) (1) exercise (*n* = 36) (2) HILT + exercise (*n* = 35) (3) Exercise + dry needling (*n* = 34)	1. No 2a. No 2b. painful area in cervical region 3. 15	1. 5/wk. 2. 3 wks	1. VAS 2. PRWE	High-intensity laser therapy	1. BTL brand 6,000 series 2. Neodymium: YAG 3. NR	1. 1,064 nm 2. 8 W 3. NR	1. 25cm^2^ 2. 125 J, 3.5 J/cm^2^ 4. 1.02 min*15 5. NR 6. 25 Hz 7. NR
Cheng et al. (53)	1. Prospective RCT 2. LBP 3. *n* = 106 (analysis *n* = 105) (1) LA + postnatal care (*n* = 53) (2) postnatal care (*n* = 52)	1. No 2a. BL23, BL25, BL26, BL40, SP6 (Bi) 2b. No 3. 10	1. 5/wk. 2. 2 wks	1. VAS 2. daily activities, physical activity, stress, cortisol, RMDQ, ODI	LA	1. Reimers and Janssen GmbH 2. GaAlAs 3. NR	1. NR 2. NR 3. NR	1. NR 2. NR 3. 4.5 J/cm^2^ 4. 5 s/point

Bi: bilateral; BL: bladder meridian; DOMS: delayed onset muscle soreness; EX-HN: extra points head and neck; RDC/TMD: Research Diagnostic Criteria for Temporomandibular Disorders SKFS: Saudi Knee Function Scale EX-LE: extra points lower extremities; GB: gall bladder meridian; HILT: high-intensity laser therapy; Hz: Hertz; J: Joule; KI: kidney meridian; LA: laser acupuncture; LBP: low back pain; LI: large intestine meridian; LILT; low-intensity laser therapy; LLLT: low-level laser therapy; LR: liver meridian; LU: lung meridian; min: minute(s); ms: millisecond(s); NMOS: neuromyo relaxing occlusal splint; NR: not reported; NRS: numerical rating scale; ns: nanosecond(s); ODI: Oswestry disability index; PC: pericardium meridian; PPT: pressure pain threshold; PRWE: patient-rated wrist evaluation; RMDQ: Roland-Morris disability questionnaire; ROM: range of motion; s: second(s); SF36: 36-Item Short Form Health Survey; SI: small intestine meridian; SP: spleen meridian; ST: stomach meridian; TE: triple energizer meridian; TMD: temporomandibular disorders; TMJ: temporomandibular joint; TP: trigger/tender points; Uni: unilateral; wk(s): week(s); WOMAC: Western Ontario and McMaster Universities Arthritis Index; VAS: visual analog scale PRWE: Patient-Rated Wrist Evaluation.

#### 3.2.2 Dosage parameters of laser acupuncture

**Figure 2:**
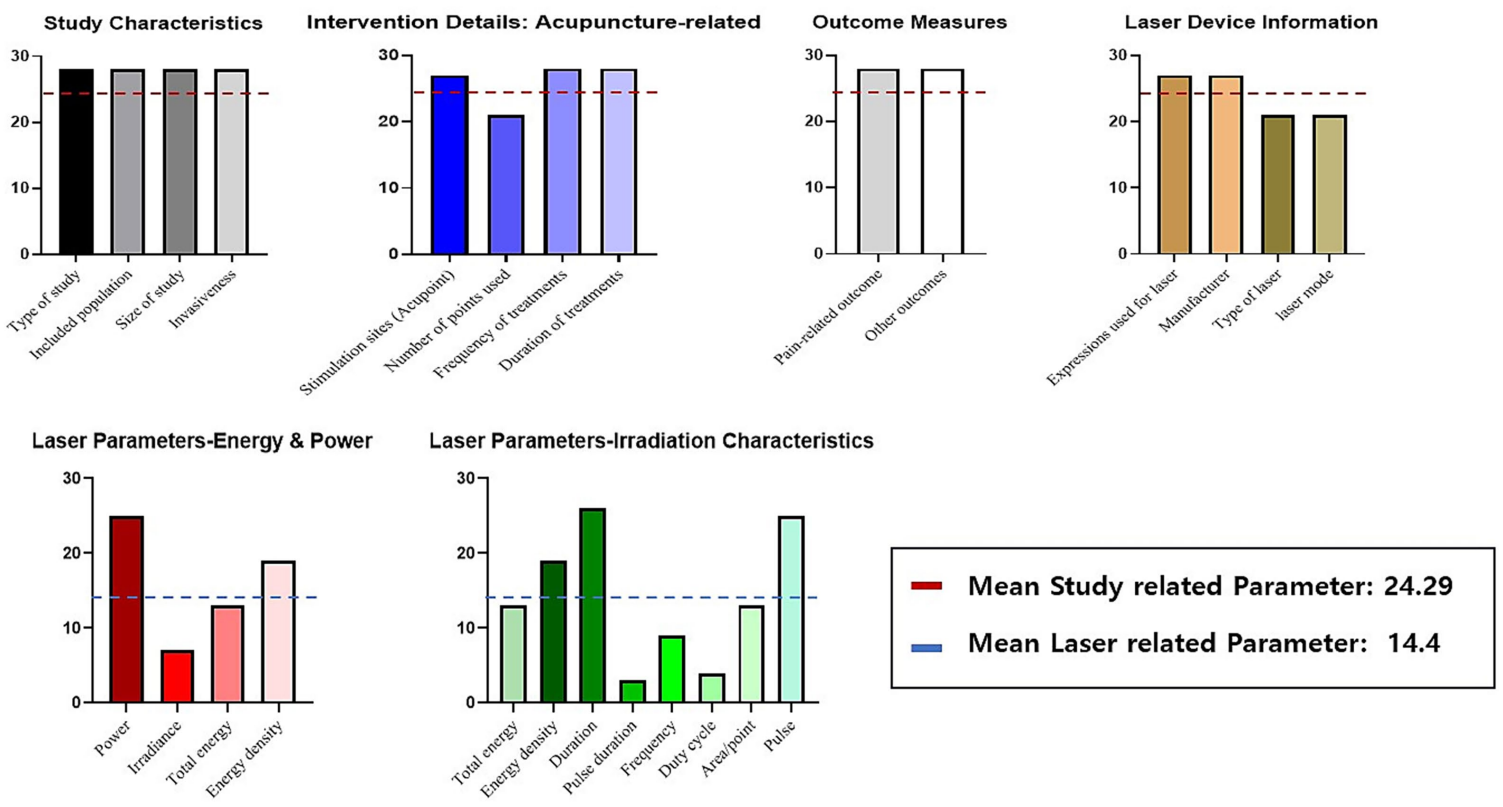
Frequency of reporting key parameters in laser acupuncture studies (number of reporting studies). This figure summarizes the reporting frequency of key parameters in 28 laser acupuncture studies, categorized into five domains: study characteristics, intervention details (acupuncture-related), outcome measures, laser device information, and laser parameters. Each bar represents the number of studies that reported a given parameter. Study-related parameters—including characteristics of the study design, details of acupuncture intervention, and outcome measures—were reported consistently across most studies, with an overall mean reporting count of 24.29 (indicated by the red dashed line). In contrast, laser-related parameters—including energy characteristics, power-related variables, and irradiation characteristics—showed substantially lower reporting frequencies, with an overall mean of 14.4 (indicated by the blue dashed line). Laser parameters were subdivided into two groups: energy and power parameters (e.g., power, irradiance, total energy, energy density) and irradiation characteristics (e.g., duration, pulse, frequency, duty cycle, area/point). These parameters exhibited the largest variability in reporting, highlighting inconsistencies in how laser characteristics are documented across studies. Overall, the figure illustrates that, while general study and acupuncture-related information is reported relatively consistently, laser-specific parameters show notable gaps and variability in reporting across the literature.

Several inconsistencies in reporting were identified among the reviewed studies. Some emphasized energy density, measured in joules per square centimeter (J/m^2^), while others focus on output power, measured in milliwatts (mW). Additionally, variability was observed in reporting treatment duration; some studies specified the irradiation time, whereas others reported only the total treatment time. Furthermore, while some studies clearly specify the type of laser, others only indicated the color of the laser. Notably, parameters such as invasiveness, number of points used, treatment duration (reported in 100% of studies), overall duration (92%), pulse and power (89%), type of laser, laser mode, and number of points used (75%) were reported in over 75% of the included studies. In contrast, parameters like the area per stimulated point, total energy (46%), and irradiance (25%) were reported in less than 50% of the studies. Similarly, irradiation-related parameters such as duty cycle and pulse duration were among the least frequently reported items, with reporting rates generally below 30%. As shown in Figure 2, these parameters exhibited substantially lower reporting frequencies compared to other laser-related variables, underscoring persistent gaps in documentation. This highlights the need for more standardized reporting practices to enhance consistency and comparability in laser acupuncture research (Table 1 and Figure 2).

#### 3.2.3 Reporting compliance with STRICTA guidelines

**Figure 3:**
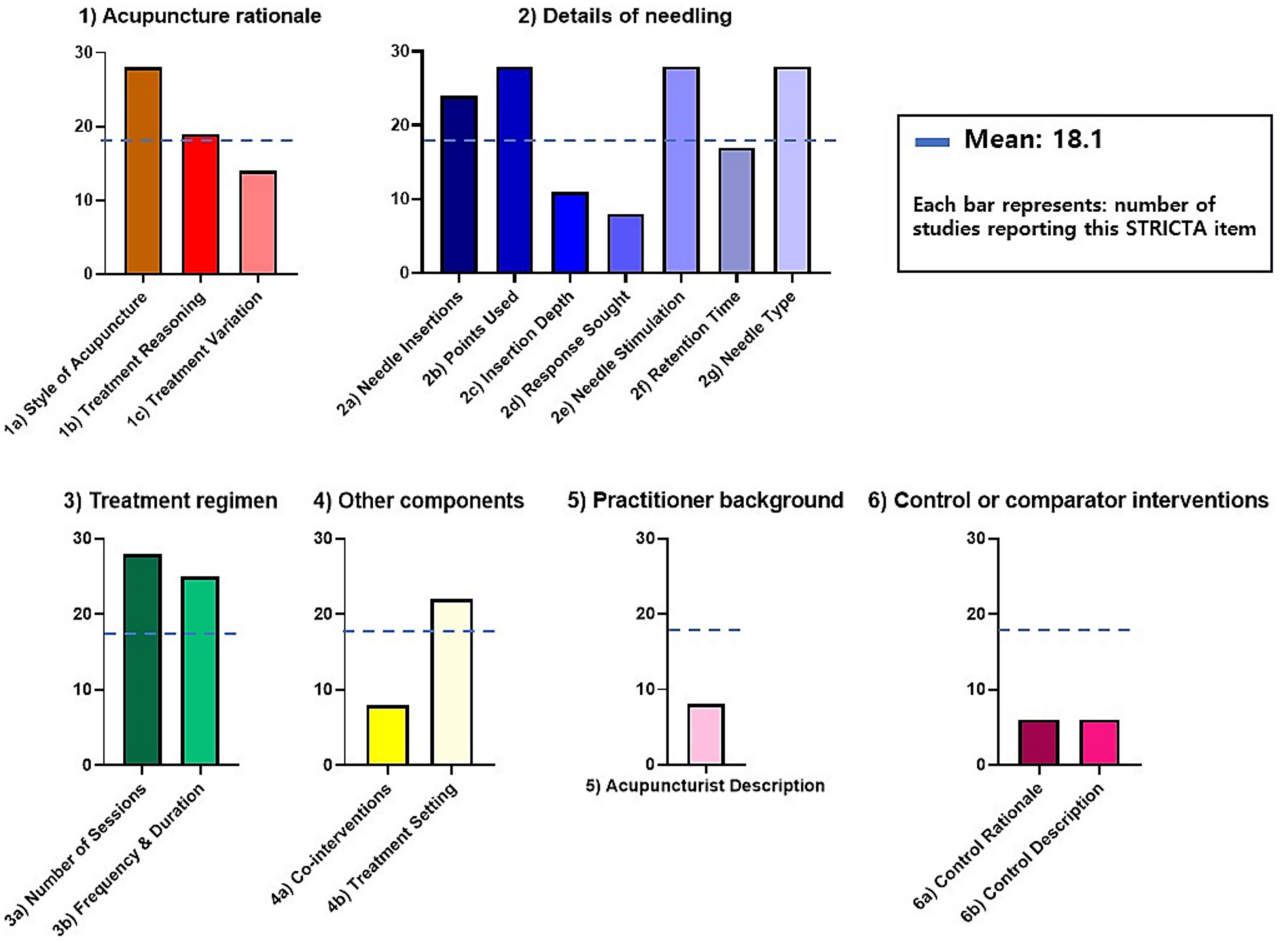
Reporting of STRICTA guidelines items in laser acupuncture studies. This figure presents the extent to which each item of the Standards for Reporting Interventions in Clinical Trials of Acupuncture (STRICTA) guidelines is reported across 28 laser acupuncture studies. The graph is organized into six major STRICTA domains—(1) Acupuncture rationale, (2) Details of needling, (3) Treatment regimen, (4) Other components, (5) Practitioner background, and (6) Control or comparator interventions. Each domain is displayed in a separate panel for clarity. Each bar represents the number of studies that reported the corresponding STRICTA item. A horizontal blue dashed line indicates the overall mean reporting count (18.1) across all items. Colors differentiate the six STRICTA domains, with panels using consistent color schemes to enhance visual grouping rather than representing performance differences. A legend is provided to clarify that each bar reflects how many of the 28 studies included that specific reporting item.

Table 2 compares the STRICTA guidelines with the proposed parameter guidelines specifically tailored for laser acupuncture, highlighting additional parameters that require consideration, such as laser manufacturer, type, mode, and specific settings including pulse duration and irradiance. An analysis of the reporting status of various parameters in studies adhering to the STRICTA guidelines revealed considerable differences in the frequency of reported parameters. For instance, parameters such as the style of acupuncture, names of the points used, needle stimulation, needle type, number of treatment sessions (reported in 100%), and frequency and duration of treatment sessions (89.3%) were reported in most studies. In contrast, parameters such as the response sought, details of other interventions, acupuncturist description (28.6%), and details of control interventions (21.4%) had relatively lower reporting rates (Figure 3 and Supplementary Table 3).

**Table 2:** STRICTA guidelines and suggested reporting guidelines for laser acupuncture.

STRICTA guidelines	Suggested reporting guideline for laser acupuncture
1. Acupuncture rationale	1. Study design
(1a) Style of acupuncture	(1a) Type of study
(1b) Reasoning for treatment provided	(1b) Included population
(1c) Extent to which treatment was varied	(1c) Size of study
2. Details of needling	2. Details of LA
(2a) Number of needle insertions	(2a) Invasiveness
(2b) Names of points used	(2b) Stimulation sites [1] Acupoints [2] Non-acupoints
(2c) Depth of insertion	(2c) Number of points used
(2d) Response sought	
(2e) Needle stimulation	
(2f) Needle retention time	
(2g) Needle type	
3. Treatment regimen	3. Treatment regimen
(3a) Number of treatment sessions	(3a) Frequency of treatment sessions
(3b) Frequency and duration of sessions	(3b) Duration of treatment sessions
4. Other components of treatment	4. Outcomes
(4a) Details of other interventions	(4a) Pain-related
(4b) Setting and context of treatment	(4b) Others
5. Practitioner background	5. Expressions used for laser
(5a) Description of participating acupuncturists	
6. Control or comparator interventions	6. Device information
(6a) Rationale for the control or comparator	(6a) Manufacturer
(6b) Precise description of the control or comparator	(6b) Type of laser
	(6c) Laser mode
	7. Device setting
	(7a) Pulse
	(7b) Power
	(7c) Irradiance
	8–1. Dose (for continuous-wave laser)
	(8-1a) Area
	(8-1b) Total energy
	(8-1c) Density
	(8-1d) Duration
	8–2. Dose (for pulsed laser)
	(8-2a) Pulse duration
	(8-2b) Frequency
	(8-2c) Duty cycles

### 3.3 Effects of laser acupuncture

**Figure 4:**
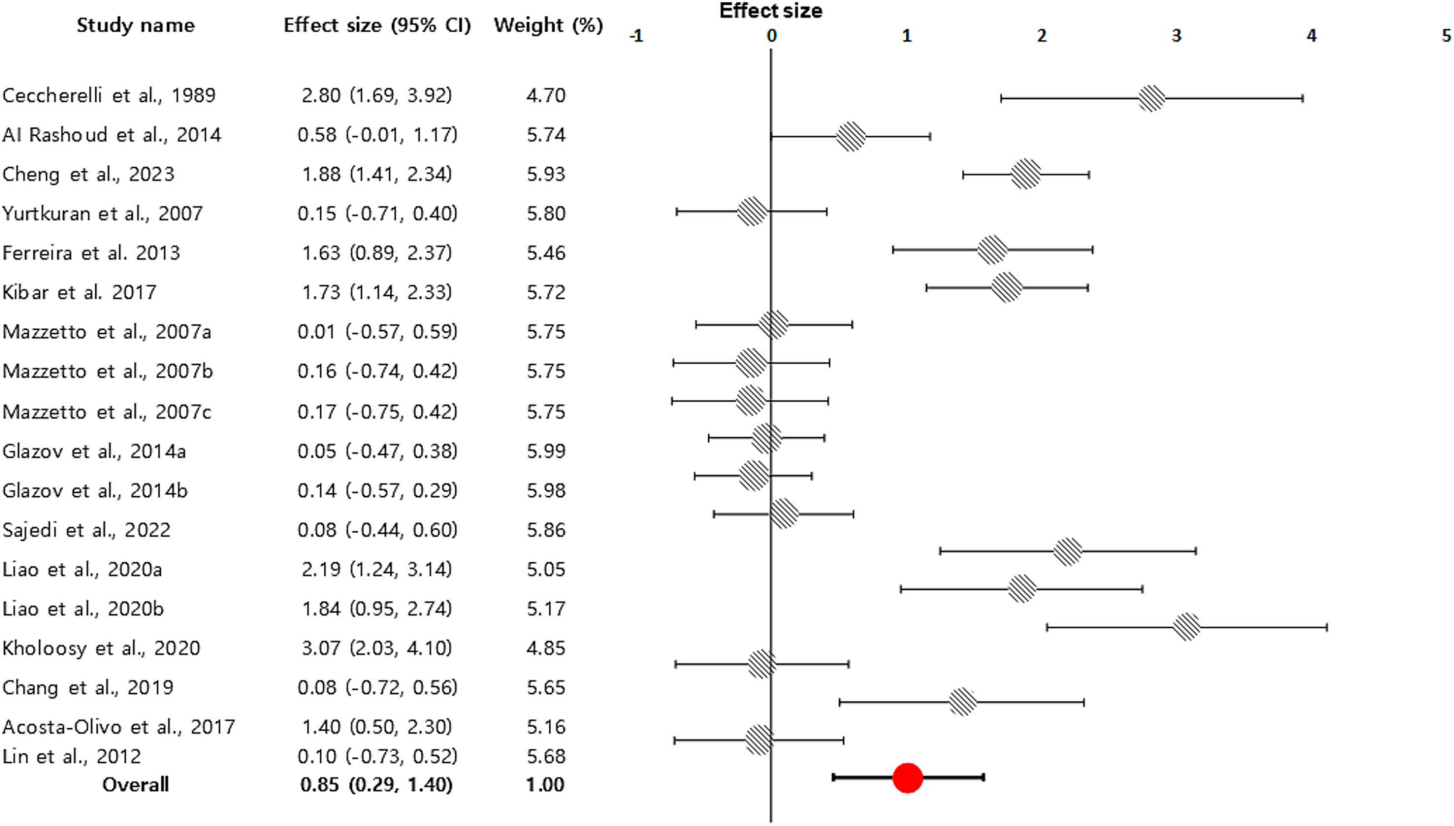
Forest plot displaying a random-effects model-based meta-analysis of the effect of laser acupuncture on various diseases. To calculate the total effect size and 95% confidence interval (CI), we used a random effects model. In 14 studies, 18 comparisons measured pain relief and demonstrated that acupuncture had a significant impact compared to the control groups (combined effect size = 0.85; 95% CI, 0.29–1.40; indicated by an orange circle with a black line).

Eighteen comparisons from 14 studies evaluated pain relief for various conditions using the VAS (*n* = 16) or the NRS (*n* = 2), demonstrating a significant effect of laser acupuncture compared to the control groups (combined effect size = 0.85; 95% CI, 0.29–1.40). Substantial heterogeneity (*I*^2^ = 90.31%) was observed. The Egger regression test indicated significant publication bias (intercept = 24.65, 95% CI, 14.50–34.79, t = 5.13, *p* < 0.001). The Begg–Mazumdar rank correlation test also indicated significant publication bias (*p* < 0.01, Figure 4). Considering these domains collectively, the overall certainty of evidence for pain reduction was judged to be low to moderate, mainly due to substantial heterogeneity, small sample sizes, risk of bias concerns in several trials, and evidence of publication bias.

## 4 Discussion

In this study, we summarized the current usage of laser acupuncture reported in RCTs targeting musculoskeletal disorders and evaluated the reporting status of various parameters. We included 28 RCTs for the systematic review and 14 studies for the meta-analysis of clinical effects of laser acupuncture. We extracted various laser parameters and examined the types and frequency of parameters reported. Although most studies detailed design-related parameters, the reporting rates for laser-related parameters were typically low (<75%). In particular, parameters essential for pulse mode lasers had low reporting rates (<30%) except for the parameter ‘Frequency.’

In this context, we used the STRICTA guidelines as an acupuncture-specific framework, analogous to general tools such as TIDieR, to structure the extraction and appraisal of intervention details in laser acupuncture trials. According to our STRICTA-based analysis, only nine out of 17 items of guideline were reported in more than the average count of 18.1 studies, highlighting a lack of detail in laser dose-related parameters. Laser acupuncture combines the therapeutic effects of lasers with the stimulation of acupoints (including traditional acupoints, Ashi points, and trigger points). However, many studies did not clearly describe the rationale for selecting acupuncture points, with a reporting rate of approximately 60% for item 1b of the STRICTA guidelines. This suggests that the rationale for acupuncture point selection has not been well reported for laser acupuncture.

Notably, there is significant inconsistency in how laser parameters were reported, with each study presenting different dosage parameters. Due to the unique characteristics of laser acupuncture, certain parameters are incompatible with the STRICTA guidelines, resulting in discrepancies in reporting. For instance, some studies reported laser dose in terms of energy density (J/cm^2^), while others focused on output power (mW). There were also inconsistencies in the reported treatment time, with variations between laser irradiation time and total treatment time. This lack of standardization complicates the comparison of clinical effects across studies.

Importantly, our findings should not be interpreted as suggesting that a single, universal stimulation protocol could be applied across all musculoskeletal disorders. Given that different conditions involve distinct pathological mechanisms, tissue characteristics, and therapeutic targets, it is more plausible that *multiple condition-specific laser acupuncture protocols* will ultimately be required. The present review does not attempt to establish such protocols; rather, by identifying inconsistencies and clarifying which parameters must be reported, our work provides the foundational evidence necessary for future studies to determine optimal, disorder-specific stimulation parameters and to develop clinical guidelines accordingly.

Because the included trials encompassed a range of musculoskeletal conditions, the pooled mean difference in pain should be interpreted as an exploratory, overall summary of the effect of laser acupuncture across musculoskeletal pain rather than as a disorder-specific estimate, and condition-specific findings are therefore also reported descriptively. This qualitative certainty assessment further indicates that current effect estimates should be interpreted with caution, and highlights the need for future rigorously designed trials with consistent parameter reporting to increase confidence in the true effect of laser acupuncture.

To address these inconsistencies in reporting, our findings highlight the need for standardized guidelines specifically tailored to laser acupuncture. Such guidelines could be developed through collaboration between researchers and clinicians and should include standardized terminology and metrics for reporting laser dosage and other critical parameters. In this study, we did not seek to establish formal guidelines; rather, we summarized parameters reported in previous RCTs and provided descriptive information that may support future consensus processes and guideline development.

The field of acupuncture has faced challenges similar to dose reporting (20, 21). The introduction of the STRICTA guidelines and research on dose–response relationships have helped mitigate these issues, improving the quality and consistency of acupuncture research. These improvements have facilitated more reliable meta-analyses and increased the number of high-quality studies. Laser acupuncture research similarly requires distinct reporting guidelines that reflect the unique characteristics of lasers. Establishing such guidelines could replicate the positive impact of the STRICTA guidelines (22, 23).

The findings of this study have important clinical implications Standardizing reporting practices may enhance the reliability of laser acupuncture research and its applicability in clinical settings by enabling clinicians and researchers to better compare study outcomes and interpret treatment effects in people with musculoskeletal disorders. Clearer reporting of stimulation parameters will also help clinicians to more confidently incorporate laser acupuncture into multimodal treatment plans.

These issues are not unique to laser acupuncture. The challenges identified in our review parallel those reported for other dose-dependent, non-invasive physical modalities. In low-level laser therapy and photobiomodulation for neuromusculoskeletal conditions, variations in parameters such as wavelength, power density, fluence and treatment time have been shown to substantially influence treatment efficacy, underscoring the complexity of dose optimization in light-based therapies (24). Similar parameter-related issues have been described for transcutaneous electrical nerve stimulation, where the choice of stimulation intensity and frequency is critical to achieving reliable analgesic effects, yet reporting of these variables remains heterogeneous across clinical trials (25). Within contemporary management of chronic musculoskeletal pain, clinical guidance increasingly emphasizes non-pharmacologic, non-invasive interventions—including exercise therapy, manual therapy and acupuncture—as part of multimodal care rather than as isolated treatments (1). Clear and consistent reporting of laser acupuncture parameters is therefore essential not only for interpreting individual trial results, but also for enabling meaningful comparison with other non-invasive approaches and for integrating laser acupuncture into evidence-based multimodal rehabilitation pathways.

However, it is important to acknowledge the limitations of this study. Our analysis was limited to the studies available at the time, which may not represent the entirety of research conducted in this field. Additionally, variability in study designs and reporting practices posed challenges in data synthesis. Because half of the included studies did not report the mean and standard deviation of pain improvements, only 14 of the 28 RCTs were included in the meta-analysis. Therefore, future studies should address these limitations by adopting uniform reporting standards and employing rigorous methodologies to ensure the generalizability of the findings. Specifically, future research should focus on conducting well-designed studies to investigate the effects of different laser acupuncture dosages and parameters, and report the parameters according to the guidelines. Clinical trials with larger sample sizes are also needed to thoroughly evaluate the dose–response relationship and explore the molecular and cellular mechanisms of laser acupuncture, which could provide valuable insights into its therapeutic potential.

## 5 Conclusion

In conclusion, this systematic review and meta-analysis suggests that laser acupuncture may provide beneficial effects for musculoskeletal disorders. However, the overall certainty of evidence is limited by methodological shortcomings, small sample sizes, and substantial heterogeneity in laser parameters across trials. Inconsistent documentation of stimulation parameters in particular hinders reliable comparison between studies and constrains confident interpretation of efficacy. To support robust clinical implementation, future research should prioritize well-designed randomized trials with clearly defined musculoskeletal populations, standardized outcome measures, and transparent, standardized reporting of laser parameters, including wavelength, power, dose, and stimulation protocol. Studies that prospectively apply and refine parameter-reporting frameworks, explore dose–response relationships, and assess long-term effectiveness and safety in real-world clinical settings will be especially valuable. Clarifying these aspects will enable clinicians to incorporate laser acupuncture more consistently into multimodal management of musculoskeletal disorders and to make more confident, evidence-based decisions in routine practice.

## Data Availability

The original contributions presented in the study are included in the article/Supplementary material, further inquiries can be directed to the corresponding author/s.
