# Supplementary material for: Laser acupuncture in the treatment of musculoskeletal disorders: systematic review and meta-analysis
Source: Front Neurol. 2026 Jan 12;16:1672380. doi: 10.3389/fneur.2025.1672380 (PMC12833518; doi:10.3389/fneur.2025.1672380)
Supplement: Supplementary file 1 [file Table_1.docx]

**Supplementary Table 1. Parameters in laser acupuncture**

| **Term** | **Description** | **Unit (Example)** |
| --- | --- | --- |
| Type of study | This refers to the research methodology (e.g., double-blind RCT, prospective RCT), and stricter study designs tend to enhance the reliability of treatment effects. | No unit |
| Included population | This indicates the condition being studied. The effectiveness of laser treatment can vary depending on the site and severity of the condition. | No unit |
| Size of study | This refers to the number of participants in the study (experimental/control group). Larger studies tend to increase the reliability of the results. | Number |
| Invasiveness | This indicates whether the treatment is invasive or non-invasive. Non-invasive treatments are generally safer for patients. | No unit |
| Stimulation sites | This indicates whether the treatment was applied to acupoints or non-acupoints, and the effectiveness of treatment can vary based on the stimulation site and the use of acupoints. | No unit |
| Number of points used | This refers to the number of stimulation points used in the treatment. The number of points and total energy are used to calculate energy per probe. | Number |
| Frequency of treatments | This indicates how manys the treatment comprised and the number ofs per wk. Higher frequency can lead to increased treatment efficacy. | Sessions/day or week |
| Duration of treatments | This refers to the total duration of the treatment. Longer therapy durations can result in better outcomes. | Days or weeks |
| Pain-related outcome | This indicates whether the reduction in pain was significant or not, marked as significant/non-significant. | - |
| Other outcomes | This indicates whether other treatment effects, apart from pain reduction, were significant or not, marked as significant/non-significant. | - |
| Expressions used for laser | This refers to the terminology used to describe the method of laser treatment, depending on the approach or technology used. Examples include PBM, LA, LLLT, LILT, HILT. | No unit |
| Manufacturer | This refers to the company that manufactured the laser device. The quality of the equipment can vary based on the manufacturer's reliability. | No unit |
| Type of laser | This indicates the type of laser used. The effectiveness can vary depending on the type of laser. | No unit |
| laser mode | This indicates whether the laser was in continuous mode or pulse mode. The impact on tissue can vary depending on the mode. | No unit |
| Pulse | This indicates the wavelength of the laser, which determines the depth of treatment and biological effects. | Nanometers (nm) |
| Power | This indicates the energy output per unit of time. Higher output power provides stronger stimulation. | (Milli)watts (mW or W) |
| Irradiance | This indicates the power output per unit area. Higher irradiance delivers more concentrated energy. | \|  \| \| --- \|  \| Watts/cm² (W/cm²) \| \| --- \| |
| Area/point | This indicates the area over which the laser is applied. Larger areas can treat wider regions. | cm² |
| Total energy | This indicates the total energy delivered. Higher total energy can lead to stronger treatment effects. | Joules (J) |
| Energy density | This indicates the total energy delivered per unit area. Higher energy density delivers more energy. | Joules/cm² (J/cm²) |
| Duration | This indicates the total time the laser is applied. Longer durations can enhance treatment efficacy. | seconds or minutes/session |
| Pulse duration | This indicates the duration of each pulse. Longer pulse durations deliver more energy to the tissue. | (Milli)seconds (ms) |
| Frequency | This indicates the frequency at which pulses occur per sec. Higher frequencies provide more stimulation instances but shorter stimulation times. | Hertz (Hz) |
| Duty cycle | This indicates the percentage of time the pulse is on. Higher duty cycles provide stronger stimulation. | Percent (%) |

Supplementary Table 2. Results of risk of bias assessment of randomized controlled clinical trials by the RoB 2.0

| **Author (year)** | **Risk by Domains** | | | | | **Overall**  **Risk of Bias** |
| --- | --- | --- | --- | --- | --- | --- |
|  | **Bias arising from the randomization process** | **Bias due to deviations from intended interventions** | **Bias due to missing outcome data** | **Bias in the measurement of the outcome** | **Bias in selection of the reported result** |  |
| (Ceccherelli, AltafiniL, Castro, Avila, Ambrosio and Giron, 1989) | High | Some concerns | Low | Low | Some concerns | High |
| (Mazzetto, Carrasco, Bidinelo, Pizzo and Mazzetto, 2007) | High | Low | Low | Low | Some concerns | High |
| (Yurtkuran, Alp, Konur, Özçakir, Bingol and therapy, 2007) | Low | Low | Low | Low | Some concerns | Some concerns |
| (Lin, Wu, Hsieh, Su, Shih, Lin, Wu and Medicine, 2012) | Some concerns | Some concerns | Low | Low | Some concerns | Some concerns |
| (Ferreira, de Oliveira, Guimarães, Carvalho and De Paula, 2013) | Some concerns | Low | Low | Low | Some concerns | Some concerns |
| (Al Rashoud, Abboud, Wang and Wigderowitz, 2014) | High | Some concerns | Low | High | Some concerns | High |
| (Glazov, Yelland and Emery, 2014) | Low | Low | Low | Low | Some concerns | Some concerns |
| (Kibar, Konak, Evcik and Ay, 2017) | High | Low | Low | Low | Some concerns | High |
| (Acosta-Olivo, Siller-Adame, Tamez-Mata, Vilchez-Cavazos, Peña-Martinez and Research, 2017) | Some concerns | Some concerns | Low | Low | Some concerns | Some concerns |
| (Chang, Wu, Chang, Lee, Chen and Medicine, 2019) | Some concerns | Low | Low | Low | Some concerns | Some concerns |
| (Liao, Lin, Lo, Chang, Liao, Chou and Medicine, 2020) | Low | Low | Low | Low | Some concerns | Some concerns |
| (Kholoosy, Elyaspour, Akhgari, Razzaghi, Khodamardi and Bayat, 2020) | Some concerns | Low | Low | Low | Some concerns | Some concerns |
| (Sajedi, Abbasi, Asnaashari and Jafarian, 2022) | Some concerns | Low | Low | Low | Some concerns | Some concerns |
| (Cheng, Wu, Tung, Shieh and Liu, 2023) | Low | Low | Low | Low | Some concerns | Some concerns |

RoB: revised Cochrane Risk of Bias Tool for Randomized Trials

**Supplementary Table 3. Adherence to STRICTA guidelines in clinical studies using laser acupuncture**

| **Author (year)** | **1. Acupuncture rationale** | **2. Details of needling** | **3. Treatment regimen** | **4. Other components of treatment** | **5. practitioner background** | **6. Control or comparator interventions** |
| --- | --- | --- | --- | --- | --- | --- |
|  | 1a) Style of acupuncture  1b) Reasoning for treatment provided  1c) Extent to which treatment was varied. | 2a) Number of needle insertions  2b) Names of points used  2c) Depth of insertion  2d) Response sought  2e) Needle stimulation  2f) Needle retention time  2g) Needle type | 3a) Number of treatments  3b) Frequency and duration of treatments | 4a) Details of other interventions administered to the acupuncture group  4b) Setting and context of treatment, including instructions to practitioners, and information and explanations to patients | 5) Description of participating acupuncturists | 6a) Rationale for the control or comparator in the context of the research question, with sources that justify this choice  6b) Precise description of the control or comparator. |
| (Madani, Ahrari, Fallahrastegar and Daghestani, 2020) | 1a) LA  1b) previous research  1c) not allowed | 2a) 6  2b) ST6, ST7, LI4 (Bi)  2c) NA  2d) NR  2e) laser  2f) 30sec  2g) laser | 3a) 10  3b) 2/wk | 4a) NR  4b) practitioner wears goggles. Participant is prohibited from taking medication. | 5) NR | 6a) NR  6b) inactive LA |
| (Aigner, Fialka, Radda and Vecsei, 2006) | 1a) LLLT  1b) Traditional acupoints, previous research  1c) not allowed | 2a) NR  2b) B10, B40, G20, G34, TW5, SI6 LG14 Earpoints 29, 37, 41, 55  2c) NA  2d) NR  2e) laser  2f) 15sec  2g) laser | 3a) 9  3b) 3/wk | 4a) Cervical collar, muscle relaxant, analgesic  4b) NR | 5) NR | 6a) NR  6b) 5mW red light lamp |
| (Chang, Tu, Pei, Chen, Wang and Wong, 2021) | 1a) LLLT  1b) previous research  1c) not allowed | 2a) 4  2b) LI10, LI4, TE5, SI3 (Uni)  2c) NA  2d) NR  2e) laser  2f) 40sec  2g) laser | 3a) 1  3b) NA | 4a) NR  4b) NR | 5) Licensed to practice acupuncture and operating the low-lever laser machine | 6a) NR  6b) inactive LA |
| (Ceccherelli, AltafiniL, Castro, Avila, Ambrosio and Giron, 1989) | 1a) laser therapy  1b) NR  1c) Acupoints + TP | 2a) 14  2b) LI4, LI11, LI14, SI3 TE5, TP (Bi)  2c) NA  2d) NR  2e) laser  2f) NR  2g) laser | 3a) 12  3b) 3/wk, 4wk | 4a) NR  4b) NR | 5) NR | 6a) NR  6b) inactive LA |
| (Shen, Zhao, Ding, Tan, Gao, Wang and Lao, 2009) | 1a) Combined LA  1b) previous research  1c) not allowed | 2a) 1 or 2  2b) ST35 (Uni-or Bi)  2c) NA  2d) NR  2e) laser  2f) 20min  2g) laser | 3a) 12  3b) 3/wk, 4 wk | 4a) NR  4b) temperature of the treatment room, description of the room, receiving treatment while wearing glasses, patient's posture | 5) Doctors | 6a) NR  6b) inactive LA |
| (Zhao, Cheng, Wu, Du, Chen, Tan, Lao and Shen, 2021) | 1a) laser moxibustion  1b) previous research  1c) Acupoint + Ashi point | 2a) NR  2b) ST35 (Uni-or Bi) ,Ashi  2c) NA  2d) NR  2e) laser  2f) 20min  2g) laser | 3a) 12  3b) 3/wk, 4 wk | 4a) NR  4b) patient's posture, Participant is allowed from taking medication. | 5) laser device operator | 6a) NR  6b) 3mW red light diode |
| (Al Rashoud, Abboud, Wang and Wigderowitz, 2014) | 1a) LLLT  1b) previous research  1c) not allowed | 2a) 5  2b) SP9, SP10, ST35, ST36, EX-LE4  2c) NA  2d) NR  2e) laser  2f) 40sec  2g) laser | 3a) 9  3b) NR | 4a) Advised to perform straight leg raise exercises  4b) posture, allowed from taking medication. | 5) NR | 6a) NR  6b) Placebo emitter |
| (Mohammed, Allam, Elghoroury, Zikri, Helmy, Elgendy and Medicine, 2018) | 1a) LA  1b) previous research, traditional asian medicine  1c) Acupoints + Ashi points | 2a) NR  2b) ST35, ST36, SP9, SP10, GB34, Ashi  2c) NA  2d) NR  2e) laser  2f) 1min  2g) laser | 3a) 12  3b) 3/wk, 4 wk | 4a) NR  4b) NR | 5) NR | 6a) NR  6b) inactive LA |
| (Cheng, Wu, Tung, Shieh and Liu, 2023) | 1a) LA  1b) previous research, TCM  1c) not allowed | 2a) 10  2b) BL23, BL25, BL26, BL40, SP6 (Bi)  2c) NA  2d) NR  2e) laser  2f) 5sec  2g) laser | 3a) 10  3b) 5/wk, 2 wk | 4a) Women postpartum  4b) standard postpartum, patient's posture | 5) TCM doctors | 6a) Elaborate description of postpartum care  6b) Only postpartum care |
| (Glazov, Schattner, Lopez and Shandley, 2009) | 1a) LA  1b) previous research  1c) Individualized | 2a) Various (average 9)  2b) Various (TP, Ashi)  2c) NA  2d) NR  2e) laser  2f) 10 or 20sec  2g) laser | 3a) 5~10 (average 9.1)  3b) 1/wk | 4a) NR  4b) general practice setting, self-helpbook (education), exercise | 5) General practitioner | 6a) NR  6b) LED |
| (Yurtkuran, Alp, Konur, Özçakir, Bingol and therapy, 2007) | 1a) LA  1b) previous research  1c) not allowed | 2a) 1  2b) SP9  2c) NA  2d) NR  2e) laser  2f) 2min  2g) laser | 3a) 10  3b) 5/wk | 4a) Exercise  4b) NR | 5) NR | 6a) NR  6b) Red light diode |
| (Ferreira, de Oliveira, Guimarães, Carvalho and De Paula, 2013) | 1a) LA  1b) previous research  1c) not allowed | 2a) NR  2b) 9  2c) NA  2d) NR  2e) laser  2f) 90sec  2g) laser | 3a) 12  3b) 1/wk | 4a) Neuro myorelaxing occlusal splint  4b) practitioner wears goggles, Bio safety protection | 5) Dentist acupuncturist | 6a) NR  6b) Sound, directed towards the regions outside of the fields of vision |
| (Kibar, Konak, Evcik and Ay, 2017) | 1a) LA  1b) previous research, TCM  1c) not allowed | 2a) 11  2b) GB21, LI4, LI11, LI14, LI15, LI16, SI9, SI10, SI11, TE14, TE15  2c) NA  2d) NR  2e) laser  2f) 40sec  2g) laser | 3a) 15  3b) 5/wk | 4a) Exercise training  4b) practitioner wears goggles | 5) Physiotherapiest | 6a) NR  6b) inactive LA |
| (Mazzetto, Carrasco, Bidinelo, Pizzo and Mazzetto, 2007) | 1a) LA  1b) previous research  1c) not allowed | 2a) 2  2b) NA  2c) NA  2d) NR  2e) laser  2f) 10sec  2g) laser | 3a) 8  3b) 2/wk | 4a) NR  4b) practitioner wears goggles, bioprotective principles | 5) Clinician | 6a) NR  6b) inactive LA |
| (Glazov, Yelland and Emery, 2014) | 1a) LA  1b) previous research  1c) Individualized | 2a) Various (average of 9)  2b) NR  2c) NA  2d) NR  2e) laser  2f) 10 or 40sec  2g) laser | 3a) Various (Maximum 8)  3b) 1/wk | 4a) NR  4b) NR | 5) Experienced general practitioner | 6a) NR  6b) inactive LA |
| (Fleckenstein, Niederer, Auerbach, Bernhörster, Hübscher, Vogt and Banzer, 2016) | 1a) LA  1b) NR  1c) Semi-standardized acupoints | 2a) NR  2b) LI4, LI11, LU3, LU5, GB34, SP10, TP, ashi + individualized  2c) NA  2d) NR  2e) laser  2f) NR  2g) laser | 3a) 3  3b) 1/day | 4a) NR  4b) practitioner wears goggles, patient's posture | 5) Expert acupuncturist | 6a) NR  6b) inactive LA |
| (Zhao, Shen, Cheng, Deng, Ding, Tan, Lao and surgery, 2010) | 1a) LA  1b) previous research  1c) not allowed | 2a) 1 or 2  2b) ST35  2c) NA  2d) NR  2e) laser  2f) NR  2g) laser | 3a) 12  3b) 3/wk | 4a) NR  4b) practitioner wears goggles, patient's posture, temperature of the treatment room | 5) NR | 6a) NR  6b) Same procedure is performed in non-acupuncture point area |
| (Sajedi, Abbasi, Asnaashari and Jafarian, 2022) | 1a) LA  1b) previous research  1c) not allowed | 2a) Various  2b) NA  2c) NA  2d) NR  2e) laser  2f) 60sec/point  2g) laser | 3a) 8  3b)3/wk | 4a) NR  4b) practitioner wears goggles, laser as instructed by the manufacturer | 5) Post-graduate student of oral medicine under the direct supervision of an oral medicine specialist | 6a) NR  6b) TP targeted soft cupping, Shamlaser |
| (Liao, Lin, Lo, Chang, Liao, Chou and Medicine, 2020) | 1a) LA  1b) Traditional acupoints, previous research  1c) not allowed | 2a) 6  2b) SP9, SP10, EX-LE2 (Bi)  2c) NA  2d) NR  2e) laser  2f) 15min  2g) laser | 3a) 12  3b) 3/wk | 4a) NR  4b) NR | 5) NR | 6a) NR  6b) inactive LA |
| (Kholoosy, Elyaspour, Akhgari, Razzaghi, Khodamardi and Bayat, 2020) | 1a) LA  1b) NR  1c) not allowed | 2a) Various  2b) LI4, ST44, H7 (Uni)  2c) 4cm  2d) NR  2e) laser  2f) 30sec/point (Non-acupoint)  10sec/point (Acupoint)  2g) laser | 3a) 12  3b) 3/wk | 4a) Naproxen  4b) NR | 5) NR | 6a) NR  6b) inactive LA |
| (Helianthi, Simadibrata, Srilestari, Wahyudi and Hidayat, 2016) | 1a) LA  1b) NR  1c) not allowed | 2a) NR  2b) ST35,ST36,SP9,GB34,EX-LE4  2c) NA  2d) NR  2e) laser  2f) 80sec/point  2g) laser | 3a) 10  3b) 2/wk | 4a) NR  4b) practitioner wears goggles and headset | 5) NR | 6a) NR  6b) inactive LA |
| (Boggiss, Rosário, de Lima, Silva, Moreira, Da Silva, De Farias, Dos Santos, Simoes and Santos, 2022) | 1a) LA  1b) previous research, Yangdorak assessment  1c) not allowed | 2a) Various  2b) LU9, PC6, HT7, LI5, TE4, SI5, SP3, LR3, Ki4, BL65, GB40, ST42 (Bi)  2c) NA  2d) NR  2e) laser  2f) NR  2g) laser | 3a) 6  3b) 2/wk | 4a) NR  4b) ventilated room, no environmental acclimatization | 5) NR | 6a) NR  6b) A health education lecture was canceled by covid-19 |
| (Chang, Wu, Chang, Lee, Chen and Medicine, 2019) | 1a) LA  1b) previous research  1c) not allowed | 2a) 2  2b) PC2, LU5  2c) NA  2d) NR  2e) laser  2f) 10min/point  2g) laser | 3a) 1  3b) 1/day | 4a) NR  4b) NR | 5) NR | 6a) NR  6b) inactive LA |
| (Ahi and Sirzai, 2022) | 1a) LA  1b) previous research, Points determined by palpation.  1c) not allowed | 2a) 15  2b) NA  2c) NA  2d) NR  2e) laser  2f) 1.02 min/point  2g) laser | 3a) 15  3b) 5/wk | 4a) Exercise program  4b) NR | 5) NR (LA) Physical medicine and rehabilitation specialist (dry needling) | 6a) NR  6b) dry needling |
| (Acosta-Olivo, Siller-Adame, Tamez-Mata, Vilchez-Cavazos, Peña-Martinez and Research, 2017) | 1a) LA  1b) previous research  1c) not allowed | 2a) 10  2b) SI5, SJ4, SJ15, LI5, PC7, BL62, BL60, KI3 (Uni) LI4 (Bi)  2c) NA  2d) NR  2e) laser  2f) 30sec/point  2g) laser | 3a) 10  3b) 3/wk | 4a) Home rehabilitation (exercises three times a day)  4b) NR | 5) NR | 6a) NR  6b) inactive LA |
| (Fang, Huang, Wang, Chen, Cheng, Deng, Lin, Zhao, Shen and Surgery, 2021) | 1a) LA  1b) previous research  1c) not allowed | 2a) Various  2b) ST35, Ashi  2c) NA  2d) NR  2e) laser  2f) 20min  2g) laser | 3a) 12  3b) 3/wk | 4a) NR  4b) NR | 5) NR | 6a) NR  6b) Sham group received traditional moxibution |
| (Lin, Wu, Hsieh, Su, Shih, Lin, Wu and Medicine, 2012) | 1a) LA  1b) previous research  1c) not allowed | 2a) Various  2b) BL40, Ashi  2c) NA  2d) NR  2e) laser  2f) 10min  2g) laser | 3a) 5  3b) 1/day | 4a) Soft cupping  4b) All patients lied down on the bed in the room air-conditioned (25◦C) and kept quiet. | 5) NR | 6a) NR  6b) Sham LA with soft cupping |
| (Haker and Lundeberg, 1990) | 1a) LA  1b) previous research  1c) not allowed | 2a) NR  2b) LI10, LI11, LI12, LU5, SJ5  2c) NA  2d) NR  2e) laser  2f) 30sec  2g) laser | 3a) 10  3b) 2-3/wk | 4a) NR  4b) NR | 5) A physician | 6a) NR  6b) red light without laser |

Bi: bilateral; BL: bladder meridian; DOMS: delayed onset muscle soreness; EX-HN: extra points head and neck; EX-LE: extra points lower extremities; GB: gall bladder meridian; HILT: high‑intensity laser therapy; Hz: Hertz; J: Joule; KI: kidney meridian; LA: laser acupuncture; LBP: low back pain; LI: large intestine meridian; LILT; low-intensity laser therapy; LLLT: low-level laser therapy; LR: liver meridian; LU: lung meridian; min: minute(s); ms: millisecond(s); NMOS: neuromyo relaxing occlusal splint; NR: not reported; NRS: numerical rating scale; ns: nanosecond(s); ODI: oswestry disability index; PC: pericardium meridian; PPT: pressure pain threshold; PRWE: patient-rated wrist evaluation; RMDQ: roland-morris disability questionnaire; ROM: range of motion; sec: second(s); SF36: 36-Item Short Form Health Survey; SI: small intestine meridian; SP: spleen meridian; ST: stomach meridian; TE: triple energizer meridian; TMD: temporomandibular disorders; TMJ: temporomandibular joint; TP: trigger/tender points; Uni: unilateral; wk(s): week(s); WOMAC: Western Ontario and McMaster Universities Arthritis Index; VAS: visual analog scale

**References**

1. Acosta-Olivo, C., Siller-Adame A., Tamez-Mata Y., Vilchez-Cavazos F., Peña-Martinez V.J.A. and Research E.-T. Laser treatment on acupuncture points improves pain and wrist functionality in patients undergoing rehabilitation therapy after wrist bone fracture. a Randomized, Controlled, Blinded Study. 42: 11-25, 2017.

2. Ahi, E.D. and Sirzai H.J.L.i.M.S. Comparison of the effectiveness of dry needling and high-intensity laser therapy in the treatment of myofascial pain syndrome: a randomized single-blind controlled study. 38: 3, 2022.

3. Aigner, N., Fialka C., Radda C. and Vecsei V.J.W.K.W. Adjuvant laser acupuncture in the treatment of whiplash injuries: a prospective, randomized placebo-controlled trial. 118: 95-99, 2006.

4. Al Rashoud, A., Abboud R., Wang W. and Wigderowitz C.J.P. Efficacy of low-level laser therapy applied at acupuncture points in knee osteoarthritis: a randomised double-blind comparative trial. 100: 242-248, 2014.

5. Boggiss, É.A., Rosário R.C., de Lima R.A., Silva P.A., Moreira R.M., Da Silva K.P., De Farias C.L., Dos Santos V.d.Q., Simoes R.P. and Santos A.T.S.J.J.o.L.i.M.S. Pulsed Laser Acupuncture in the Treatment of Pain and Heart Rate Variability in Fibromyalgia Patients: A Pilot Randomized Clinical Trial. 13: 2022.

6. Ceccherelli, F., AltafiniL L., Castro G.L., Avila A., Ambrosio F. and Giron G.J.T.C.j.o.p. Diode laser in cervical myofascial pain: a double-blind study versus placebo. 5: 301-304, 1989.

7. Chang, W.-D., Wu J.-H., Chang N.-J., Lee C.-L., Chen S.J.E.B.C. and Medicine A. Effects of laser acupuncture on delayed onset muscle soreness of the biceps brachii muscle: a randomized controlled trial. 2019: 6568976, 2019.

8. Chang, W.-H., Tu L.-W., Pei Y.-C., Chen C.-K., Wang S.-H. and Wong A.M.J.B.j. Comparison of the effects between lasers applied to myofascial trigger points and to classical acupoints for patients with cervical myofascial pain syndrome. 44: 739-747, 2021.

9. Cheng, H.-Y., Wu B.-Y., Tung T.-H., Shieh C. and Liu C.-T.J.P.M.N. Laser acupuncture analgesia on postpartum low back pain: A prospective randomized controlled study. 24: 89-95, 2023.

10. Fang, J., Huang Z., Wang X., Chen L., Cheng K., Deng H., Lin L., Zhao L., Shen X.J.P., Photomedicine, and Surgery L. Comparison of 10.6 μm laser moxibustion with traditional moxibustion in knee osteoarthritic therapy: a randomized noninferiority clinical trial. 39: 492-498, 2021.

11. Ferreira, L.A., de Oliveira R.G., Guimarães J.P., Carvalho A.C.P. and De Paula M.V.Q.J.L.i.m.s. Laser acupuncture in patients with temporomandibular dysfunction: a randomized controlled trial. 28: 1549-1558, 2013.

12. Fleckenstein, J., Niederer D., Auerbach K., Bernhörster M., Hübscher M., Vogt L. and Banzer W.J.C.j.o.s.m. No effect of acupuncture in the relief of delayed-onset muscle soreness: results of a randomized controlled trial. 26: 471-477, 2016.

13. Glazov, G., Schattner P., Lopez D. and Shandley K.J.A.i.M. Laser acupuncture for chronic non-specific low back pain: a controlled clinical trial. 27: 94-100, 2009.

14. Glazov, G., Yelland M. and Emery J.J.A.i.M. Low-dose laser acupuncture for non-specific chronic low back pain: a double-blind randomised controlled trial. 32: 116-123, 2014.

15. Haker, E. and Lundeberg T. Laser treatment applied to acupuncture points in lateral humeral epicondylalgia. A double-blind study. *Pain* 43: 243-247, 1990.

16. Helianthi, D.R., Simadibrata C., Srilestari A., Wahyudi E.R. and Hidayat R.J.A.M.I. Pain reduction after laser acupuncture treatment in geriatric patients with knee osteoarthritis: a randomized controlled trial. 48: 114-121, 2016.

17. Kholoosy, L., Elyaspour D., Akhgari M.R., Razzaghi Z., Khodamardi Z. and Bayat M.J.J.o.l.i.m.s. Evaluation of the therapeutic effect of low level laser in controlling low back pain: a randomized controlled trial. 11: 120, 2020.

18. Kibar, S., Konak H.E., Evcik D. and Ay S.J.P.M. Laser acupuncture treatment improves pain and functional status in patients with subacromial impingement syndrome: a randomized, double-blind, sham-controlled study. 18: 980-987, 2017.

19. Liao, F.-Y., Lin C.-L., Lo S.-F., Chang C.-C., Liao W.-Y., Chou L.-W.J.E.B.C. and Medicine A. Efficacy of Acupoints Dual‐Frequency Low‐Level Laser Therapy on Knee Osteoarthritis. 2020: 6979105, 2020.

20. Lin, M.-L., Wu H.-C., Hsieh Y.-H., Su C.-T., Shih Y.-S., Lin C.-W., Wu J.-H.J.E.B.C. and Medicine A. Evaluation of the effect of laser acupuncture and cupping with ryodoraku and visual analog scale on low back pain. 2012: 521612, 2012.

21. Madani, A., Ahrari F., Fallahrastegar A. and Daghestani N.J.L.i.m.s. A randomized clinical trial comparing the efficacy of low-level laser therapy (LLLT) and laser acupuncture therapy (LAT) in patients with temporomandibular disorders. 35: 181-192, 2020.

22. Mazzetto, M.O., Carrasco T.G., Bidinelo E.F., Pizzo R.C.d.A. and Mazzetto R.G.J.C. Low intensity laser application in temporomandibular disorders: a phase I double-blind study. 25: 186-192, 2007.

23. Mohammed, N., Allam H., Elghoroury E., Zikri E.N., Helmy G.A., Elgendy A.J.J.o.C. and Medicine I. Evaluation of serum beta-endorphin and substance P in knee osteoarthritis patients treated by laser acupuncture. 15: 20170010, 2018.

24. Sajedi, S.M., Abbasi F., Asnaashari M. and Jafarian A.A.J.G.M.J. Comparative Efficacy of Low-Level Laser Acupuncture and Cupping for Treatment of Patients with Myofascial Pain Dysfunction Syndrome: A Double-blinded, Randomized Clinical Trial: Comparison of the Effects of LLL Acupuncture and Cupping. 11: 1, 2022.

25. Shen, X., Zhao L., Ding G., Tan M., Gao J., Wang L. and Lao L.J.L.i.m.s. Effect of combined laser acupuncture on knee osteoarthritis: a pilot study. 24: 129-136, 2009.

26. Yurtkuran, M., Alp A., Konur S., Özçakir S., Bingol U.J.P. and therapy l. Laser acupuncture in knee osteoarthritis: a double-blind, randomized controlled study. 25: 14-20, 2007.

27. Zhao, L., Cheng K., Wu F., Du J., Chen Y., Tan M.T., Lao L. and Shen X.J.T.J.o.r. Effect of laser moxibustion for knee osteoarthritis: a multisite, double-blind randomized controlled trial. 48: 924-932, 2021.

28. Zhao, L., Shen X., Cheng K., Deng H., Ding G., Tan M., Lao L.J.P. and surgery l. Validating a nonacupoint sham control for laser treatment of knee osteoarthritis. 28: 351-356, 2010.
